# Stem cell homing in periodontal tissue regeneration

**DOI:** 10.3389/fbioe.2022.1017613

**Published:** 2022-10-13

**Authors:** Lingxi Meng, Yige Wei, Yaxian Liang, Qin Hu, Huixu Xie

**Affiliations:** ^1^ State Key Laboratory of Oral Diseases, Department of Head and Neck Oncology Surgery, National Clinical Research Center for Oral Diseases, West China Hospital of Stomatology, Sichuan University, Chengdu, China; ^2^ Faculty of Dentistry, The University of Hong Kong, Hong Kong, China

**Keywords:** periodontal regeneration, endogenous stem cell, cell homing, regenerative medicine, stimulation strategies

## Abstract

The destruction of periodontal tissue is a crucial problem faced by oral diseases, such as periodontitis and tooth avulsion. However, regenerating periodontal tissue is a huge clinical challenge because of the structural complexity and the poor self-healing capability of periodontal tissue. Tissue engineering has led to advances in periodontal regeneration, however, the source of exogenous seed cells is still a major obstacle. With the improvement of *in situ* tissue engineering and the exploration of stem cell niches, the homing of endogenous stem cells may bring promising treatment strategies in the future. In recent years, the applications of endogenous cell homing have been widely reported in clinical tissue repair, periodontal regeneration, and cell therapy prospects. Stimulating strategies have also been widely studied, such as the combination of cytokines and chemokines, and the implantation of tissue-engineered scaffolds. In the future, more research needs to be done to improve the efficiency of endogenous cell homing and expand the range of clinical applications.

## 1 Introduction

Periodontal tissue is a kind of complex tissue composed of both soft tissue like periodontal ligament (PDL) and gingiva, as well as hard tissue such as alveolar bone and cementum. It plays a crucial role in supporting teeth, bearing occlusal forces, and maintaining oral mucosa integrity. When faced with the destruction of periodontal tissue, negative effects such as tooth loss, and physical and mental health damage could happen (Darveau, 2010; Kinane et al., 2017). The existing clinical treatment methods, such as guided tissue/bone regeneration (GTR/GBR) can improve the clinical efficacy of various tissue defects (Bottino et al., 2012; Xu et al., 2019). However, the effect on periodontal regeneration is not as satisfying, especially in the recovery of physiological structure and function. The ideal result of periodontal therapy is to regenerate the cementum-periodontium-bone system while achieving this goal remains a huge challenge. However, the insufficiency of stem/progenitor cells seems to be the primary limitation for periodontal membrane and alveolar bone reconstruction.

With the development and improvement of cell therapy, researchers have found various stem cells for periodontal regeneration. Mesenchymal stem cells (MSCs) and periodontal ligament stem cells (PDLSCs) are most studied, together with other stem cells such as dental pulp stem cells (DPSCs) and induced pluripotent stem cells (iPSC) (Huang et al., 2009b; Kwon et al., 2018). Those stem cells are routinely isolated, cultured, and amplified *in vitro* and relatedly applied. However, exogenous stem cell transplantation still has various disadvantages, for example, the complicated technology of *in vitro* operation, high-cost *ex vivo* cell culture, and even the potential ethical and safety risks (Lee et al., 2016; Pacelli et al., 2017; Safina and Embree, 2022).

Stem cell homing is a physiologic process which been studied since the 1970s. The fact that intravenously injected HSPCs can find their way home to the marrow was first found in clinical transplant settings. To address the shortcomings of cell transplantation, researchers turned to the homing of endogenous stem cells, attempting to heal wounds by activating the self-repairing capacity by recruiting endogenous stem cells to the defect area (Chen et al., 2010; Ahmed et al., 2021). Stem-cell-homing involves a series of physiological processes including cell recognition, migration, proliferation, and differentiation, and ultimately achieves tissue regeneration, which plays a huge role under certain conditions and has achieved remarkable therapeutic effects (Liesveld et al., 2020). In this method, biomaterials were utilized for bioactive factors delivery as well as the host’s inherent regenerative potential activating (Safina and Embree, 2022; Xin et al., 2022; Yao and Lv, 2022; Zhao et al., 2022). By mobilizing appropriate stem/progenitor cells to specific spaces for tissue repair through cell-material interactions at the defect site, the endogenous regeneration process can be mimicked (Andreas et al., 2014; Mao et al., 2022). The possible advantages of this strategy in promoting periodontal regeneration are as follows: firstly, it provides a solution to some of the limitations of stem cell transplantation, and transforms periodontal regeneration treatment methods into a clinically valid way; secondly, it gives full play to the potential of host self-repair and regeneration, making periodontal tissue regeneration safer; moreover, compared with the introduction of exogenous stem cells, it is simpler and less expensive to treat periodontal diseases and other diseases (Abdulghani and Mitchell, 2019; Safina and Embree, 2022). To make better use of the stem cell homing technique to restore the periodontal defect, this paper reviews the research status of the stem cell homing technique.

## 2 Brief history of cell homing for tissue engineering

Stem cell homing was originally defined as the process of endothelialization through blood vessels and migration of hematopoietic stem cells (HSCs) after transplantation, and finally, HSCs colonize in the bone marrow and restart hematopoiesis, in which many cytokines and chemokines are involved (Ji et al., 2015; Zhu et al., 2015). Blood lineages are considered to be risen from hematopoietic stem and progenitor cells (HSPCs), which then migrate to the bone marrow niche through homing for further proliferation and differentiation (Li et al., 2018a; Blokzijl-Franke et al., 2021; Ranzoni et al., 2021). The homing mechanism of HSPCs is relevant to stem cell transplantation therapy and has been investigated by many researchers (Morrison and Scadden, 2014; Li et al., 2018a; Liesveld et al., 2020), though the specific mechanism of HSPCs homing was not elucidated. While researchers considered that specific cells or cytokines may mediate this process, thus enabling the homing of HSCs (Li et al., 2018a).

**FIGURE 1:**
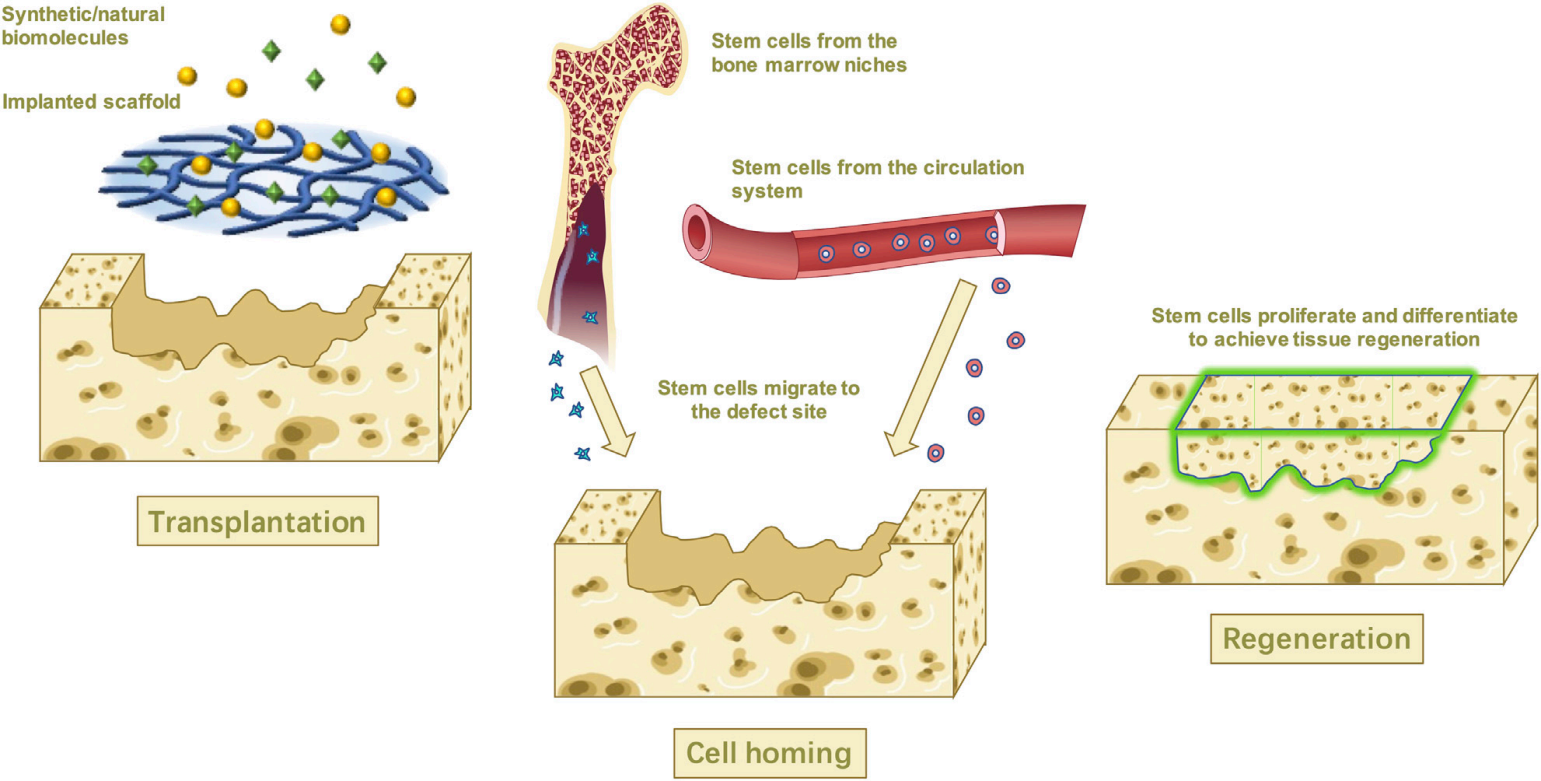
*In situ* tissue engineering uses biological molecular and scaffolds to recruit the stem cells from the niches while promoting proliferation and differentiation and achieving tissue regeneration in the end.

Recently, the tissue engineering technique has expanded the meaning of stem cell homing as the process in which endogenous stem cells mainly migrate directionally and across the vascular endothelium to target tissues, and then colonize and survive (Ji et al., 2015; Zhu et al., 2015). *In vivo*, stem cells are located in various stem cell niches and are exposed to a large number of complex and manageable biomaterials, including chemokines, cytokines, growth factors, the extracellular matrix (ECM), and so on (Chen et al., 2011; Yang et al., 2020) (Figure 1).

Tissue regeneration, also known as *in situ* tissue engineering, has emerged over the past 20 years because of its remarkable advantages. This technique mimics the human wound healing process and aims to repair or regenerate tissue by recruiting endogenous stem cells to the defections *via* targeted specific bionic scaffolds and/or bioactive cues that stimulate the host’s biological substance and repairing capacity (Ji et al., 2015; Safina and Embree, 2022). To make the endogenous stem cells migrate toward a specific tissue, these endogenous stem cells must recognize the biomolecules that mediate homing first and manipulate their activity to catalyze the homing process (Chen et al., 2011). The possible mechanism might rely on the development of the scaffold system which is implanted into the damaged area (Lee et al., 2016; Abdulghani and Mitchell, 2019). The proper microenvironment created by the implanted scaffold will then promote the host stem cells to recruit and move through the vascular network or tissue interstitial space to the damaged tissue or organ, where the stem cells then proliferate, differentiate, and form tissue within the scaffold which eventually degrades, leaving only regenerated tissue (Zheng et al., 2019; Chu et al., 2022).

In regenerative medicine, there is growing evidence proving that cell recruitment could stimulate self-repair ability in hosts and harness the born regenerative capacity of tissues. It was regarded as a promising cell-based therapy and has been used to regenerate heart tissue, cartilage tissue, and bone tissue *in situ* already (Chen et al., 2011; Cai et al., 2018). In terms of articular cartilage repair, it has been demonstrated that intra-articular and peri-articular MSCs are involved in the cartilage repair process, but due to their limited number, they are unable to achieve the desired repair effect and require stem cells from other sites to recruit to the injured site for repair and to facilitate this process, chemotactic agents are required (Yang et al., 2020; Zhang et al., 2020). Lee et al. (2010)utilized poly-ε-caprolactone and hydroxyapatite to create an anatomically correct TGF-β3-containing biological scaffold. The results suggest that for such complex tissues, the regeneration may happen *via* endogenous cell homing instead of cell transplantation. In addition, endogenous stem cell homing technology has shown great potential in the cardiovascular field, aiming at repairing heart-damaged tissues in myocardial infarction (MI) and ischemic heart disease, and is considered one of the most promising therapeutic strategies (Pacelli et al., 2017; Li et al., 2018b). Shafiq et al. (2018) developed specific cardiac patches, which can promote the mobilization and recruitment of endogenous MSCs to the defected area in acute MI models. The experimental results showed that this new cardiac patch together with suitable scaffold materials is a good choice for promoting *in situ* vascular regeneration and deserves to be promoted.

For periodontal regeneration, though the injured periodontium possesses a weak ability for self-healing, it can be significantly promoted when proper treatment and guidance are added (Xu et al., 2019). Increasing evidence demonstrates that directing endogenous stem cells to defected areas contributes to the regenerative and immunomodulating function since the resident MSCs play a key role in periodontal regeneration. Except for MSCs, PDL also contains PDLSCs and osteogenic progenitor cells which can regenerate cementum, bone, and the PDL tissue itself together with MSCs (Cai et al., 2018; Yamamoto et al., 2018; Xu et al., 2019). Additionally, a variety of factor-loaded scaffolds for periodontal regeneration have been fabricated. Yu et al. (2022) constructed a biphasic scaffold combining intrafibrillarly mineralized collagen (IMC) and concentrated growth factor (CGF) to synergist regeneration of periodontal tissues. In animal experiments, PDLSCs, BMSCs, and induced pluripotent stem cells showed the potential to stimulate the formation of new periodontal tissues (Ji et al., 2015). Consequently, the application of cell homing could eliminate clinical constraints associated with periodontal wounds (Cai et al., 2018). Wang et al. (2016) constructed a cell-free stromal cell-derived factor-1α (SDF-1α)-scaffold-parathyroid hormone system which can stimulate the proliferation of CD90^+^CD34^−^stromal cells and promote the regeneration of damaged tissues in a rat periodontal defect model. Liang et al. (2021) also used rats to apply SDF-1 topically and exendin-4 (EX-4) systemically. The experimental results showed that combined SDF-1/EX-4 treatment could promote the recruitment of MSCs *in vivo*, induce early osteoclastogenesis, and promote the expression of osteogenic proteins, which results in both the quantity and quality improvement of regenerated bone. Although the cell homing for periodontal regeneration is still at the experimental stage, it has great potential to facilitate bone-ligament-cementum regeneration in the treatment of periodontal diseases, such as periodontitis, providing a safe, effective and cost-effective alternative therapy (Liu et al., 2019a; Xu et al., 2019).

## 3 Main strategies for the regeneration of periodontal tissues by endogenous stem cells homing

**FIGURE 2:**
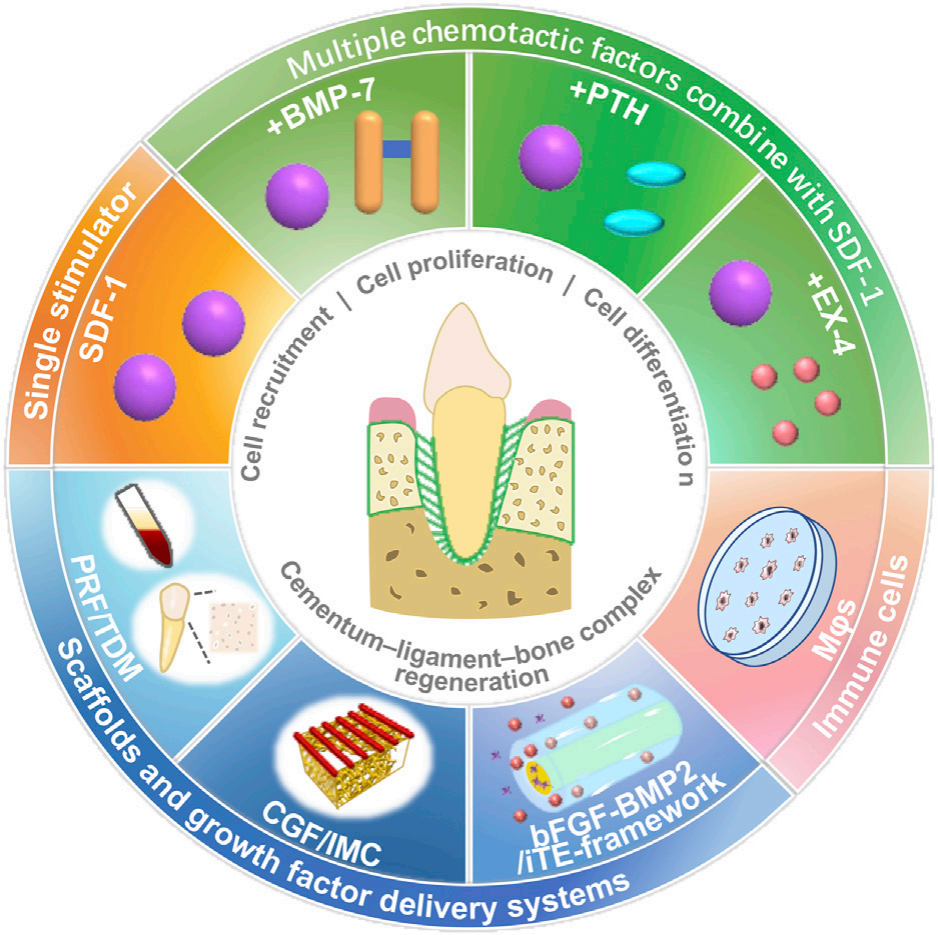
Stimulation strategies, such as chemokines, cytokines, scaffolds, and immune cells, for stem cell homing and periodontal tissue regeneration.

In periodontal tissue regeneration and repair, endogenous stem cells mainly used include BMSCs and PDLSCs, which can migrate to the defect site and stimulate their proliferation and differentiation by adopting appropriate strategies, such as chemokines, and using tissue-engineered scaffolds (Figure 2; Table 1).

**TABLE 1:** Stem cells and the stimulation strategies in the research for periodontal regeneration.

Stem cells	Recruitment stimulation	Proliferation stimulation	Differentiation stimulation	Scaffolds	*In vivo* model	Regeneration periodontal tissues	References
PDLSCs	SDF-1	—	BMP-7	Neutralized type I collagen solution	Beagle dog tooth avulsion models	PDL tissue	Zhu et al. (2015)
MSC/HSC	SDF-1	—	—	Absorbable collagen membranes	Rat mandibular bone defect models	Periodontal bone	Liu et al. (2015)
CD90+CD34−stromal cells	PTH/SDF-1	—	—	Medical collagen repair membranes	Rat periodontal defect models	Bone, cementum and functional PDL	Wang et al. (2016)
BMSCs	SDF-1	—	BMP-7	An artificial scaffold made from PCL and HA hybrid	Rat orthotopic/ectopic tooth regeneration models	PDL and new bone	Kim et al. (2010)
PDLSCs	SDF-1	—	EX-4	Medical collagen membranes	Rat periodontal defect models	Periodontal bone	Liang et al. (2021)
BMSCs	SDF-1	—	IL-4/Mφs	High-stiffness TG-gels	Rat periodontal defect models	Bone, cementum and soft tissue	He et al. (2019)
BMSCs/PDLSCs	PRF	PRF	PRF/TDM	Fabrication of canine TDM and PRF membrane	Beagle dog periodontal defect models	Cementum–PDL complex and tooth root	Ji et al. (2015)
BMSCs/PDLSCs	CGF/IMC	CGF/IMC	CGF/IMC	Hierarchical CGF/IMC bilayer architecture	Rat periodontal defect models	Bone, cementum and PDL	Yu et al. (2022)
BMSCs	SDF-1	—	—	Commercially available gelatin sponge	Rat periodontal defect models	PDL and new bone	Cai et al. (2018)
PDLSCs/MSCs	bFGF/iTE-framework	bFGF/iTE-framework	BMP-2/iTE-framework	iTE-framework	Rat periodontal defect models	Bone, cementum and PDL	Ding et al. (2020)

### 3.1 The sources of endogenous stem cell

To repair periodontal tissues, reparative cells can be acquired from stem niches (Kimura et al., 2014; Liu et al., 2015; Yin et al., 2017). Mainly BMSCs and PDLSCs were studied. PDLSCs are the first choice for studying cell delivery for periodontal regenerative purposes, while bone marrow-derived cells also contribute to it because of the insufficiency of the stem cells in PDL tissue (Yin et al., 2017).

#### 3.1.1 MSCs derived from bone marrow

Multipotent MSCs can be proliferated both *in vitro* and *in vivo*, which then differentiate into mesodermal tissues of different functions, including bone, cartilage, tendons, fat, and nerves (Pittenger et al., 1999). As a consequence, MSCs have been proposed as potentially useful tissue-engineering seeding cells. Several studies have found that BMSCs can be the origin of cementoblasts, osteoblasts, and periodontal fibroblasts, in addition to secreting extracellular matrix from PDLs, cementum, and alveolar bone (Kawaguchi et al., 2004; Hasegawa et al., 2006).

Immunohistochemistry was used to quantify the engraftment of MSCs in the defect by Liu et al. (2015). A sequential section approach was used to identify MSCs (CD29+/CD45−), which showed that the number of MSC transplanted with SDF-1 significantly increased.

He et al. (2019) selected rat BMSCs to investigate Mφ polarization and endogenous stem cell recruitment which enhance periodontal regeneration. It has been shown that the homing of BMSCs was significantly improved both *in vitro* and *in vivo*.

#### 3.1.2 PDLSCs from odontogenic tissues

Recently, subsequent attempts were made to harvest MSCs from odontogenic tissues, such as periodontal ligaments (Seo et al., 2004), gingiva (Zhang et al., 2009), the dental follicle (Morsczeck et al., 2005), the dental pulp (Gronthos et al., 2000), apical papilla (Sonoyama et al., 2008), and human exfoliated tissue deciduous teeth (Miura et al., 2003; Hynes et al., 2012). From human-impacted third molars, Seo et al. (2004) successfully isolated PDLSC, which can differentiate into multiple periodontal tissues. (Liu et al., 2008; Huang et al., 2009a; Park et al., 2011).

PDLSCs, located around the blood vessels of periodontal tissues and having the characteristics of mesenchymal stem cells, are one of the most practically applied multipotency stem cells in the field of periodontal tissue repair and regeneration. Liang et al. (2021) tested PDLSCs and confirmed that the proliferation and migration of PDLSCs. In other research, the effects of growth factors on controlling the fate of PDLSCs are studied (Ding et al., 2020).

#### 3.1.3 Non-hematopoietic stromal cells derived from hosts

CD90^+^CD34^−^ stromal cells have also been studied for recruitment. CD90, also referred to as Thy-1, represents stem and progenitor cells on the cell surface, which makes CD90^+^CD34^−^ stromal cells to be considered non-hematopoietic stromal cells. A previous study also showed that MSCs, including PDLSCs, could express CD73, CD90, and CD105 while not CD14, CD34, and CD45 (Dominici et al., 2006). Therefore, according to previous studies, CD90^+^CD34^−^stromal cells were considered as MSCs (Wang et al., 2016).

Several studies have shown that adult stem cells with CD90 expression have high osteogenic differentiation potential (Chung et al., 2013). In the study by Wang et al. (2016), stromal cells with CD90^+^CD34^−^ staining were analyzed quantitatively using immunofluorescence double staining. The results showed that host-derived CD90^+^CD34^−^stromal cells were recruited and transplanted into the defect. Ding et al. (2020) also used the framework to recruit CD90^+^CD34^−^ stromal cells. The *in vivo* research results showed significant promotion of the recruitment of host-derived CD90^+^CD34^−^ stromal cells at the early stage of wound healing, favoring tissue repair and regeneration.

#### 3.1.4 Multiple sources derived stem cells

Some researchers studied the recruiting and regulating fate of PDLSCs and BMSCs together (Ji et al., 2015; Yu et al., 2022). According to Ji et al. (2015), PDLSCs and BMSCs were tested *in vitro* with canine platelet-rich fibrin (PRF) and treated dentin matrix (TDM). An orthotopic transplantation model using Beagle dogs was developed to regenerate roots with a tooth-PDL-alveolar interface by cell homing in a canine orthotopic model, combined with the use of PRF membranes and TDM. *In vitro*, PRF significantly stimulated the recruitment and proliferation of PDLSCs and BMSCs. The *in vivo* results similarly illustrated that roots connected to the alveolar bone by cementum-PDL complexes can be regenerated by implanting PRF and TDM in the alveolar microenvironment, possibly through the homing of BMSCs and PDLSCs.

### 3.2 The strategies of stem cell homing

Currently, the main chemotactic strategies used include: 1) the use of chemotactic agents, such as sdf-1, and the combination of the two; 2) the use of scaffold materials loaded with biological factors; 3) regulatory assistance through macrophages. They are described as follows:

#### 3.2.1 Local application of SDF-1

SDF-1, also known as C–X–C motif ligand 12 (CXCL12), is a promising candidate for *in situ* tissue engineering among various cytokines and chemokines. Previous studies have shown that during the healing process of impaired tissues [brain (Ardelt et al., 2013), heart (Wang et al., 2012; Hajjar and Hulot, 2013; Cavalera and Frangogiannis, 2014), muscle (Brzoska et al., 2012), skin (Yang et al., 2013), and bone (Ji et al., 2013)], elevated levels of SDF-1 at the site of injury can recruit stem/precursor cells from the cardiovascular system and local tissues, and promote their proliferation and differentiation at the site of damage, leading to organ repair and regeneration (Brzoska et al., 2012; Wang et al., 2012; Hajjar and Hulot, 2013; Yang et al., 2013).

In the study of Liu et al. (2015), it has been verified that SDF-1 promoted the proliferation, migration, and differentiation of PDLSCs *in vitro*. After that, they further confirmed the effect of local application of SDF-1 on stem/progenitor cell homing and periodontal bone regeneration *in vivo* through a rat mandibular defect model. Host-derived CD29+/CD45− MSCs were confirmed to be triggered to home and graft into periodontal bone defects by loading SDF-1 into collagen membrane scaffolds. As a result of SDF-1 treatment, MSCs were significantly increased and CD45 + HSCs were engrafted.

The same results were obtained by Adelina S. (Cai et al., 2018) using an SDF-1α loaded gelatin sponge (Spongostan^®^) for rats with mandibular bone defects. At the same time, a possible explanation has been proposed regarding the enhanced periodontal regeneration caused by SDF-1α stimulation: first, bone marrow-derived osteogenic progenitor cells present in the flowing blood may be recruited to the defected area by the SDF-1α/CXCR4 axis in response to the local release of SDF-1α (Otsuru et al., 2008; Ji et al., 2013). The recruited cells play an important role in periodontal regeneration by expressing their multilineage differentiation capacity, as well as secreting promoting cytokines and growth factors (Bryan et al., 2005). Furthermore, SDF-1α may also induce other progenitor cells in the bone marrow such as HSCs and endothelial progenitor cells to migrate to the defect sites *via* the SDF-1α/CXCR4 axis, creating a proangiogenic environment (Hattori et al., 2003; Petit et al., 2007).

#### 3.2.2 SDF-1 in combination with other chemotactic factors

The main effect of SDF-1 is to actively direct endogenous cell homing. However, its promoting effect on the proliferation and differentiation of stem cells is limited. Therefore, combining other active molecules, such as bone morphogenetic protein 7 (BMP-7), parathyroid hormone (PTH), and EX-4, to improve the efficiency of tissue regeneration has been studied.

BMP-7 could modulate osteogenesis and bone cell differentiation potently. A preclinical study assessed its effects on the regeneration of periodontal bone defects and improvement of cementum regeneration (Cook et al., 1995; Giannobile et al., 1998; Ripamonti et al., 2001). Groundbreaking research into the regeneration of PDL tissue *via* cell homing with SDF1 and BMP-7 was carried out by Kim et al. (2013) in 2010. BMP-7 may be responsible for orthotopic mineralization and formation of the newly formed alveolar bone in rat extraction sockets. The observed putative periodontal ligament also suggests that SDF1 and/or BMP-7 can recruit multiple cell lineages. On this basis, Zhu et al. (2015) further studied the application of SDF1 and BMP-7 in the tooth trauma model frequently. Zhu et al. (2015) hypothesized that SDF1 and BMP-7 may recruit endogenous cells to the root surface to replace the unavailable original PDL cells after avulsed teeth were delayed. During the experiment, avulsed roots coated with SDF1 and BMP-7 were put into the prepared alveolar bone socket. By directing stem cells to the space between replanted roots and the adjacent alveolar bone, SDF1 and BMP-7 were proven to establish the integrated PDL-like structure.

PTH has been used as an optional treatment for bone defects. In periodontal tissue repair, when a large number of endogenous progenitor cells are mobilized directly to the peripheral blood, they will return to the defect site and participate in tissue regeneration, along which PTH is considered a promising periodontal tissue repair agent (Barros et al., 2003; Bashutski et al., 2010; Tokunaga et al., 2011). As a crucial factor for stem cell homing, the chemotactic properties of SDF-1α may be limited by the N-terminal cleavage of the cell surface protein CD26/dipeptidyl peptidase-IV (DPP-IV) at position 2 proline (Christopherson et al., 2002; Christopherson et al., 2004). PTH, known as a DPP-IV inhibitor, has been confirmed recently to enhance the SDF-1α-driven homing of CXCR4 + stem cells in the ischemic heart recently (Huber et al., 2011). In a rat periodontal defect model, Wang et al. (2016) confirmed that PTH/SDF-1α cotherapy was able to induce CD90 + CD34^−^stromal cell migration and enhance the chemotactic ability of SDF-1α, and accelerate periodontal tissue regeneration.

EX-4, as a full agonist of the glucagon-like peptide-1 receptor (GLP-1R), could promote both migration and proliferation of MSCs (Zhou et al., 2015; Zhang et al., 2016; Sharma et al., 2018; Yap and Misuan, 2019). Recently, studies on EX-4 have proved its capability to inhibit adipogenic stem/precursor cell differentiation while promoting osteogenic differentiation and bone formation. (Feng et al., 2016; Meng et al., 2016; Luciani et al., 2018). Moreover, the quantity of CXCR4+ MSCs was increased by EX-4 *via* PI3K/AKT-SDF-1/CXCR4 signaling pathway (Zhou et al., 2015). Liang et al. (2021) demonstrated that the combination of SDF-1/EX-4 enhanced the proliferation and migration of PDLSCs *in vitro*, together with the recruitment of MSCs, inducement of early osteoclastogenesis, expression of osteoblast protein in new bone formation, as long as the formation of new bone *in vivo*. Therefore, the combination of SDF-1/EX-4 could provide a new strategic option for periodontal bone regeneration *in situ*.

#### 3.2.3 Chemotaxis effect of scaffold and its role as growth factor carriers

Scaffold biomaterials should be designed to mimic the natural extracellular environment as much as possible to affect cell behavior and control cell fate *in vivo*. In the present study, many scaffold materials can not only act as carriers but also play a role in recruiting stem cells and promoting their proliferation and differentiation.

Choukroun et al. (2001) reported in 2001 that PRF could promote the recruitment, proliferation, and differentiation of stem cells by releasing several growth factors, such as transforming growth factor-β(TGF-β), platelet-derived growth factor (PDGF), epidermal growth factor (EGF). TDM, a natural acellular matrix scaffold, could retain essential non-collagenous proteins and growth factors for the regeneration of apical periodontal tissue (Li et al., 2008; Smith et al., 2012). Both PRF and processed TDM are depots of various growth factors that can promote cell homing. Ji et al. (2015) confirmed that PRF can recruit BMSCs and PDLSCs. In addition, the proliferation of PDLSCs and BMSCs was also stimulated *in vitro*, which is consistent with other studies (Ehrenfest et al., 2010; Chang and Zhao, 2011). The results also demonstrated that TDM was able to direct the differentiation of seed cells (Ma et al., 2008; Wu et al., 2008; Li et al., 2011; Guo et al., 2012; Yang et al., 2012). Treated as one unit, PRF/TDM’s potential to stimulate the differentiation of PDLSCs and BMSCs was confirmed *in vitro*. *In vivo*, the experimental results showed that it is essential for endogenous tooth root regeneration by using PRF as bioactive cues, and TDM as an inductive scaffold, together with tooth socket microenvironments.

In addition to scaffold materials of biological origin, synthetic scaffold materials also have similar effects. Ding et al. (2020) developed a super assembly framework (SAF) in which bFGF and BMP-2 were designed to facilitate regeneration of the local cementum-ligament-bone complex in a sequential manner. The in *situ* tissue engineering framework (iTE-framework) not only showed improved physicochemical properties, but also was shown to promote the proliferation, migration, and osteogenic differentiation of PDLSCs *in vitro*. A rat periodontal defect model is created for *in vivo* experiment. The results showed that both the formation of new bone and the regeneration of PDL and cementum tissue could be promoted by the iTE framework significantly.

#### 3.2.4 The role of immunomodulation in stem cell recruitment

Microenvironments can influence cell homing by influencing the properties of stem cells. Since there are already many well-established drugs targeting the immune microenvironment in treating periodontitis, it is a promising strategy to combine these existing therapeutic agents and cytokines to enhance the immune microenvironment and promote cell homing and tissue formation, thus achieving higher levels of immune regulation and tissue repair (Yang et al., 2021).

In correlative studies (Abbott et al., 2004), exogenous SDF-1 was found to be insufficient to stimulate stem cell recruitment without injury for periodontal regeneration. A combination of inflammation and SDF-1 may increase stem cell recruitment in the healing process (Chen et al., 2013). In further studies conducted by Li et al. (2018a), it was confirmed that VCAM-1 + macrophage-like cells are important for both homing and retention of HSPCs. In the field of periodontal defect regeneration, He et al. (2019) studied the effect of “macrophage regulation” in periodontal tissue regeneration. It was hypothesized that high-stiffness TG gels modulate Mφ polarization and promote endogenous stem cell recruitment by modulating IL-4 and SDF-1α production. Immunofluorescence staining and histological examination showed that IL-4 could promote the polarization of Mφs into the M2 phenotype, and further promote the osteogenic differentiation of successfully homed BMSCs.

In the future, further studies on the role of immunomodulation in stem cell homing are needed to address these uncertain issues and reach scientific conclusions.

### 3.3 Scaffolds contributing to stem cell regulation

#### 3.3.1 Natural scaffold materials

Natural scaffold materials have good biocompatibility and bioactivity, and can also be loaded with a variety of biological factors, effectively promoting the reconstruction of periodontal tissues.

Collagen, as a natural protein, is obtained from the skin, bones, and ligaments of animals and is widely used in tissue engineering scaffolds due to its superior biocompatibility, biodegradability, and weak antigenicity (Reyes and García, 2004; Xiong et al., 2009). In the study of recruiting endogenous stem cells for periodontal tissue regeneration, collagen membrane or collagen solution is often used as a tissue engineering scaffold and plays a role in transporting chemokines and recruiting endogenous stem cells (Liu et al., 2015; Zhu et al., 2015; Wang et al., 2016; Liang et al., 2021). A commercial product, gelatin sponge (Spongostan ^®^) (Cai et al., 2018), was also used as a carrier for the delivery of SDF-1α.

TDM is a kind of natural matrix scaffold that is decellularized and retains multiple bioactive molecules, including non-collagenous proteins and growth factors (Smith et al., 2012) that are crucial for the formation of periodontal tissue around tooth roots (Li et al., 2008). Several studies have shown that PDLSCs treated with dentin non-collagenous proteins exhibit several signs of differentiation into cementoblasts. In their previous studies, TDM demonstrated a role as an inductive microenvironment and scaffold for tooth root regeneration (Guo et al., 2012; Yang et al., 2012). TDM, compared with nature dentin, has similarities in structure and mechanical characteristics but is more cost-effective. By regulating molecules in a mechanically-suitable environment, it is expected to trigger regenerative processes not only structurally but also physiologically. There was evidence that TDM can direct the differentiation of seeded cells (Ma et al., 2008; Wu et al., 2008; Li et al., 2011; Guo et al., 2012; Yang et al., 2012). The study by Ji et al. (2015) mentioned above demonstrated the effect of TDM in periodontal tissue regeneration.

Another scaffold material of natural origin was prepared by Yu et al. (2022). With biomimetic self-assembly and microstamping techniques, they constructed a parallel hierarchy of mineralized IMC layers and unmineralized collagenized-CGF layers. With special micro-structure, mechanical characteristics, and growth-factor-rich microenvironment, the differential biphasic scaffold simulates the periodontal hard/soft tissue interface perfectly. This solves the obvious disadvantage that monophasic scaffolds cannot be used effectively for regenerating complex multiphasic tissues, such as periodontal tissues (Dan et al., 2014; Cho et al., 2016). This biomimetic IMC scaffold modulates and determines the fate of PDLSC and BMSCs. Even though it cannot secrete growth factors, the osteoid microenvironment can serve as a biofactor-rich microenvironment that facilitates cell osteogenesis (Liu et al., 2019b). CGF is rich in endogenous growth factors, including TGF-β1, VEGF, PDGF-BB, IGF-1, and bFGF. The CGF substrate superstructure with parallel-aligned gaps may also be responsible for the oriented migration of stem cells (Liu et al., 2013). *In vitro* experiments demonstrated that PDLSCs were successfully differentiated into PDL/osteogenesis using the biphasic scaffold containing CGF/IMC. After implantation in rat periodontal defects, multilineage differentiation could be effectively achieved by recruiting host stem cells into soft and hard periodontal tissues.

#### 3.3.2 Composite scaffolds

Natural scaffold materials have good biocompatibility and bioactivity, but synthetic scaffold materials usually have more controllable degradation rates and physical and mechanical properties. To overcome the disadvantages of the above materials when used alone, as well as simulate complex periodontal tissues, a new direction in the development of scaffold materials in recent years is to combine two or more materials to obtain good physical and chemical properties while improving their biocompatibility and bioactivity.

Unlike previous approaches that relied primarily on hard materials like collagen, silk, or PLGA, the primary scaffold relies on soft materials (Young et al., 2002; Modino and Sharpe, 2005; Ikeda et al., 2009). The mechanical stiffness of polycaprolactone (PCL) and hydroxyapatite (HA) hybrid has good load-bearing properties (Woodfield et al., 2005). Rapid prototyping methods such as 3D bioprinting may offer additional advantages such as precise control, interconnectivity, as well as anatomical dimensions (Woodfield et al., 2005; Lee et al., 2009). Kim et al. (2010), anatomically shaped human layer scaffold and rat incisor scaffold from a hybrid of PCL and HA *via* 3D layer-by-bioprinting channels. The collagen solution loaded with SDF1 and BMP-7 was also injected into the scaffold microchannel by a micropipette. Next, the rat model was used for orthotopic/ectopic tooth regeneration without cell transplantation. Quantitatively, the combination of SDF1/BMP-7 significantly improved the cell’s chances of homing into the microchannels of human molar scaffolds compared to scaffolds without growth factors. Besides recruitment of cells into the microchannels, the regeneration of putative PDL and new alveolar bone also supports the claim that cell homing is effective.

Other composite scaffolds, such as a three-dimensional (3D) scaffold of high-stiffness Transglutaminase cross-linked gelatin (TG-gel) constructed by He et al. (2019) and framework (SAF) which can sequentially release bFGF and BMP-2 constructed by Ding et al. (2020), also showed great potential for *in situ* periodontal tissue regeneration.

## 4 Application of endogenous stem cells homing in periodontal tissue regeneration

Periodontal tissue is a complex tissue composed of PDL, alveolar bone, cementum, and gingiva, which together play an important role in supporting teeth, masticatory forces, and maintaining the mucosa of the oral masticatory. In addition to controlling the inflammation in the periodontal tissues caused by periodontitis (Pihlstrom et al., 2005; Darveau, 2010; Kinane et al., 2017), periodontal therapy aims to regenerate the cementum-ligament-bone complexes (Phillips et al., 2000). The traditional method is stem cell transplantation (Liu et al., 2008), which is unsatisfactory for a complete and stable restoration of periodontal tissue. The use of *in situ* tissue engineering for periodontal regeneration has recently gained popularity. Specifically, scaffolds are used to deliver chemotactic agents to maximize the host’s intrinsic potential, mobilize appropriate progenitor cells into an area designated for tissue repair, and mimic endogenous regenerative processes (Chen et al., 2015; Martino et al., 2015; Lee et al., 2017). Therefore, the method can regenerate and repair a variety of periodontal tissues, including periodontal ligaments, alveolar bone, cementum, and periodontal ligament fibers (Table 1).

### 4.1 Hard tissue (alveolar bone and cementum) regeneration

In severe cases of periodontitis, the alveolar bone could be resorbed, resulting in tooth loss. During periodontal therapy, the main goal is to regenerate bone. Tissue engineering techniques have opened up new possibilities for regenerating periodontal bone. Liu et al. (2015) performed *in vitro* experiments using a Wistar rat model of mandibular buccal bone defects. The effects of SDF-1 on bone formation were assessed. At the early stage of degradation, old bone was resorbed through osteoclastogenesis facilitated by SDF-1, and collagen scaffold formation was accelerated by MMP-9, making space for new bone and other tissue. The anti-inflammatory properties of SDF-1 could reduce the inflammatory response, promote vascularization, recruit MSCs and HSCs to the wound for healing processes, and ultimately enhancing bone regeneration. Even though it is still unclear which mechanism governs bone regeneration, the study confirms that loading SDF-1 into collagen scaffolds shows great potential as *in situ* tissue engineering strategy in periodontal bone regeneration.

It is insufficient to apply SDF-1 alone for favorable bone regeneration (Cipitria et al., 2017). Hence, other growth factors should be combined to boost periodontal bone regeneration. An *in vitro* and *in vivo* study conducted by Liang et al. (2021) showed that SDF-1/EX-4 combination treatment enhanced PDLSC proliferation and migration, as well as *in vitro* mineral deposition production and early osteoclastogenesis. The osteogenic protein expression in a rat periodontal bone defect model has also been upregulated. This strategy therefore improves the quantity and quality of regenerated bone and provides a new tool for periodontal bone regeneration *in situ*.

### 4.2 Soft tissue (periodontal ligament) regeneration

In dental trauma, tooth avulsion is one of the most common but severe cases (Andreasen et al., 1994). After the tooth was avulsed, the fibers of its PDL were torn, which would render immediate but severe injury to the periodontal soft tissue. To survive after avulsed teeth are replanted, regeneration of PDL is essential.

By coating the root surface with SDF-1 and BMP-7, Wenting Zhu et al. (2015) succeeded in generating a PDL-like neo-tissue between the surrounding alveolar bone and the replanted root surface. Since the collagen fibers were inserted deep into the cementum and adjacent bone perpendicularly, neo-tissue displayed periodontium-like characteristics. In essence, it was possible to restore the integrity of the periodontal structure of the teeth. Using this method, it would be possible to determine whether avulsed teeth could be rescued after they had been given up clinically.

### 4.3 Reparation of complex multiphase periodontal tissues

The disease of periodontitis involves both periodontal ligaments and alveolar bone, which causes inflammation of gingival tissues, the loss of periodontal attachment, and ultimately leads to tooth loss (Pihlstrom et al., 2005). To limit inflammation within the periodontal tissues and control the progression of periodontitis, the main goal of periodontal therapy is to restore these lost tissues to their original morphology, structure, and function. Due to the complex mineralized/non-mineralized layered structure of periodontal tissues (Woo et al., 2021), regenerative repair is a challenging task for dentists (Bartold et al., 2000) once the loss of hard tissue and soft tissue occurs. While stem cell-based cell delivery therapy shows great promise in periodontal wound healing, culture-expanded stem cells require complex procedures and are expensive to apply. (Chen et al., 2010; Chen et al., 2012b). Endogenous regeneration techniques can stimulate potential self-repair mechanisms in the host by promoting endogenous stem cell recruitment and adaptation to the lesion site (Chen et al., 2011). Compared to complex and expensive *ex-vivo* manipulation techniques, these options enhanced safety, affordability, and flexibility, making them increasingly popular in periodontal regenerative medicine (Chen et al., 2010).

Using the rat periodontal fenestration defects model, Wang et al. (2016) explored the effects of a cell-free system that combined PTH systemic application to SDF-1α-scaffold on periodontal tissue regeneration *in vivo*, while co-presentation of IL -4 and SDF-1α in high-stiffness TG-gels on periodontal regeneration was studied by He et al. (2019). Two experiments both led to satisfactory soft tissue and hybrid tissue regeneration.

**FIGURE 3:**
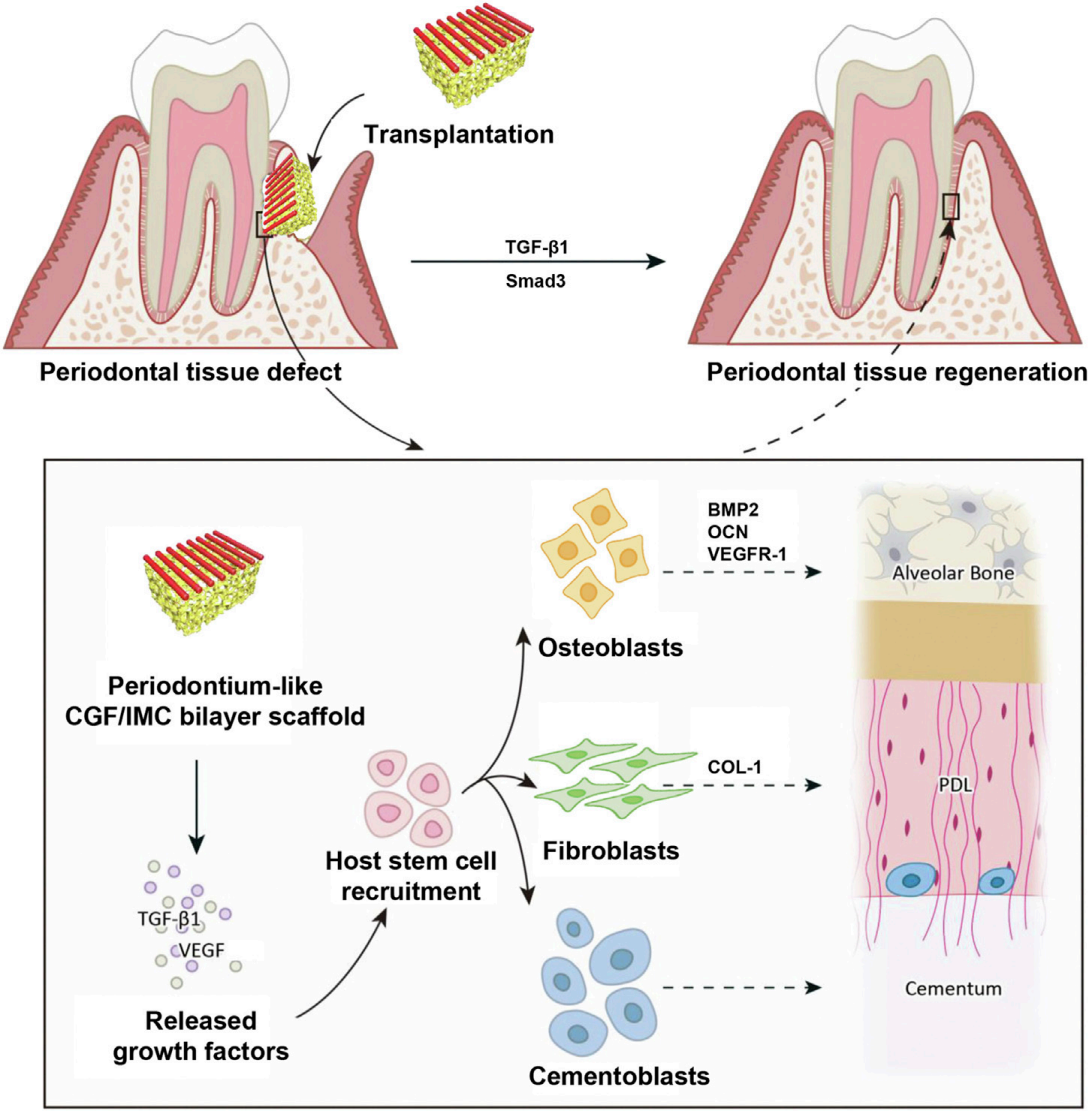
Potential mechanisms of periodontal hard/soft tissue regeneration by the hierarchical CGF/IMC bilayer architecture. Reprinted from ref (Yu et al. (2022)).

Due to the difficulty of regenerating complex multiphase tissues by monophasic scaffolds (Dan et al., 2014; Cho et al., 2016), Yu et al. (2022) constructed a biomimetic tissue-specific functional structure to regenerate periodontal tissues (Figure 3). In the hierarchical biphasic architecture, IMC scaffolds could be used for osteogenic differentiation, while CGF fibers exhibited fibroblastic differentiation potential. After implanting the critical-sized intact defect in a rat model, the host stem cells could be recruited effectively, which then achieve multilineage differentiation into periodontal tissues. As the PDL fibers were inserted into the newly formed bone tissue, the CGF/IMC biphasic scaffold successfully reconstructed complete and functional periodontium after 8 weeks of implantation.

## 5 Conclusion and prospects

The repair of periodontal defects is a difficult problem of international concern. The existing traditional clinical treatment methods, such as GTR and GBR, fail to restore the physiological structure and function of teeth and periodontal tissues effectively. With the rapid advancements in cell therapy, stem cell transplantation has become the main focus and means of promoting tooth and periodontal regeneration. However, *in vitro* culture of stem cells requires strict conditions, complex procedures, and faces a huge risk of clinical application. The use of *in vivo* endogenous stem cell migration to promote tissue regeneration is expected to solve the difficulties of stem cells *in vitro* and reduce the risk of clinical application, which has a broad research prospect and clinical application space.

Stem cell homing involves a series of physiological processes such as cell adhesion, migration, proliferation, and remodeling, and requires appropriate scaffold materials as well as a certain microenvironment. Current research mainly includes *in vitro* cell experiments and *in vivo* animal experiments, while does not involve clinical trials yet. At present, the chemotactic strategies mainly use a variety of chemotactic agents, such as SDF-1, BMP-7, EX-4, PRF, to encourage the migration and proliferation of endogenous stem cells, as well as the differentiation into periodontal tissues. Some scaffold materials are also involved in chemotaxis. Additionally, the application of immune cells (such as macrophages) in cell homing has also been studied.

However, despite the validated role of stem cell homing in periodontal tissue regeneration, there are still significant limitations of the strategy. Firstly, the stem cell sources are hard to define, and the mechanisms by which chemotactic strategies promote stem cell homing have not been fully elucidated. Additionally, the property of endogenous stem cells may be affected under certain physiological conditions such as inflammation and aging, which will limit the effect of stem cell homing strategy. On the other hand, the currently used animal models are comparatively limited and lack *in vivo* findings in mammals. Meanwhile, due to the maturity of the technology itself, it has not been put into clinical trials.

In the future, the regulation of endogenous stem cell fate by different chemotactic agents and the combination of chemotactic agents and scaffold materials will remain the key point of research. Further understanding of homing mechanisms and development of methods to enhance this phenomenon may have further clinical benefits. At the same time, more advanced detection techniques are required to identify the source of recruited stem cells. In addition, improving the animal experimental model of periodontal tissue engineering is also the focus of future research. Only by clarifying the mechanism and obtaining reliable results on a suitable animal model can the transformation from basic experiments to clinical applications be achieved as soon as possible.
